# Benzyl 2-(4-bromo­anilino)-4,4-dimethyl-6-oxo­cyclo­hex-1-enecarbodithio­ate: second triclinic polymorph

**DOI:** 10.1107/S1600536809006151

**Published:** 2009-02-25

**Authors:** El Sayed H. El Ashry, Mohammed R. Amer, M. Raza Shah, Seik Weng Ng

**Affiliations:** a H.E.J. Research Institute of Chemistry, International Center for Chemical and Biological Sciences, University of Karachi, Karachi 75270, Pakistan; b Department of Chemistry, University of Malaya, 50603 Kuala Lumpur, Malaysia

## Abstract

The title structure, C_22_H_22_BrNOS_2_, is a triclinic modification. Whereas the other reported modification crystallizes with just one mol­ecule in the asymmetric unit, the present modification has *Z*′ = 2. The six-membered cyclo­hexene ring adopts an envelope conformation, with the C atom bearing the two methyl groups representing the flap. This atom deviates by 0.674 (4) Å from the plane passing through the other five atoms of the ring (r.m.s. deviation = 0.027 Å). For the second independent mol­ecule, the deviation is 0.669 (3) Å and the r.m.s. deviation is 0.010 Å. The mol­ecular conformation of both mol­ecules is stabilized by intra­molecular N—H⋯S hydrogen bonds.

## Related literature

For background and the other triclinic modification of C_22_H_22_BrNOS_2_, see: El Ashry *et al.* (2009 ▶).
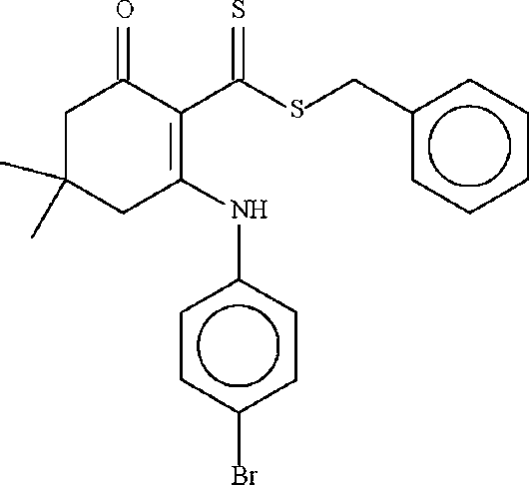


## Experimental

### 

#### Crystal data

C_22_H_22_BrNOS_2_
*M* *_r_* = 460.44
Triclinic, 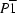

*a* = 11.8210 (2) Å
*b* = 12.8904 (2) Å
*c* = 15.8833 (3) Å
α = 67.670 (1)°
β = 71.434 (1)°
γ = 73.283 (1)°
*V* = 2083.56 (6) Å^3^
*Z* = 4
Mo *K*α radiation
μ = 2.19 mm^−1^
*T* = 100 K
0.40 × 0.15 × 0.05 mm

#### Data collection

Bruker SMART APEX diffractometer
Absorption correction: multi-scan (*SADABS*; Sheldrick, 1996 ▶) *T*_min_ = 0.667, *T*_max_ = 0.899
19892 measured reflections
9548 independent reflections
7113 reflections with *I* > 2σ(*I*)
*R*_int_ = 0.029

#### Refinement

*R*[*F* ^2^ > 2σ(*F* ^2^)] = 0.034
*wR*(*F* ^2^) = 0.091
*S* = 1.00
9548 reflections
499 parameters
2 restraints
H atoms treated by a mixture of independent and constrained refinement
Δρ_max_ = 0.64 e Å^−3^
Δρ_min_ = −0.80 e Å^−3^

### 

Data collection: *APEX2* (Bruker, 2008 ▶); cell refinement: *SAINT* (Bruker, 2008 ▶); data reduction: *SAINT*; program(s) used to solve structure: *SHELXS97* (Sheldrick, 2008 ▶); program(s) used to refine structure: *SHELXL97* (Sheldrick, 2008 ▶); molecular graphics: *X-SEED* (Barbour, 2001 ▶); software used to prepare material for publication: *publCIF* (Westrip, 2009 ▶).

## Supplementary Material

Crystal structure: contains datablocks global, I. DOI: 10.1107/S1600536809006151/bt2877sup1.cif

Structure factors: contains datablocks I. DOI: 10.1107/S1600536809006151/bt2877Isup2.hkl

Additional supplementary materials:  crystallographic information; 3D view; checkCIF report

## Figures and Tables

**Table 1:** Hydrogen-bond geometry (Å, °)

*D*—H⋯*A*	*D*—H	H⋯*A*	*D*⋯*A*	*D*—H⋯*A*
N1—H1⋯S2	0.90 (2)	2.11 (2)	2.890 (2)	146 (2)
N2—H2⋯S4	0.88 (2)	2.10 (2)	2.887 (2)	149 (2)

**Fig. 1.**
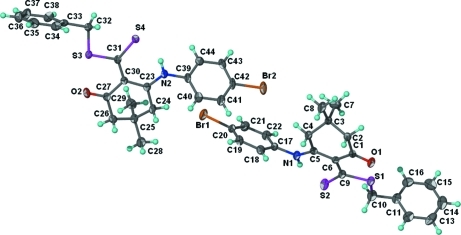
Anisotropic displacement ellipsoid plot (Barbour, 2001) plot of the two indepedent molecules of C22H22BrNOS2 at the 70% probability level; hydrogen atoms are drawn as spheres of arbitrary radius.

## supplementary crystallographic information

### Experimental

To a solution of 3-(4-bromoanilino)-5,5-dimethyl-cyclohex-2-en-1-one (0.1 mol) in DMSO (20 ml) and sodium hydroxide (0.1 mol) in water (1 ml) was added carbon disulphide (0.3 mol). The mixture was kept at 263 K for 20 min. Benzyl bromide (0.1 mol) was added. The mixture was left for 24 h, after which it was quenched with water (200 ml) and then acidified with 10% hydrochloric acid. The resulting precipitate was collected by filtration, dried and purified on a silica gel column (30% ethyl acetate in hexane) to give orange crystals (40% yield).

### Refinement

Carbon-bound H-atoms were placed in calculated positions (C—H 0.93 to 0.99 Å) and were included in the refinement in the riding model approximation, with *U*(H) set to 1.2 to 1.5*U*(C). The methyl groups were allowed to rotate but not to tip. The amino H-atoms were located in a difference Fourier map, and were refined with a distance restraint of N–H 0.88±0.01 Å; their   isotropic displacement parameters were freely refined.

### Crystal data

**Table:**

C_22_H_22_BrNOS_2_	*Z* = 4
*M**_r_* = 460.44	*F*(000) = 944
Triclinic, *P*1	*D*_x_ = 1.468 Mg m^−^^3^
Hall symbol: -P 1	Mo *K*α radiation, λ = 0.71073 Å
*a* = 11.8210 (2) Å	Cell parameters from 5387 reflections
*b* = 12.8904 (2) Å	θ = 2.3–28.2°
*c* = 15.8833 (3) Å	µ = 2.19 mm^−^^1^
α = 67.670 (1)°	*T* = 100 K
β = 71.434 (1)°	Prism, orange
γ = 73.283 (1)°	0.40 × 0.15 × 0.05 mm
*V* = 2083.56 (6) Å^3^	

### Data collection

**Table:**

Bruker SMART APEX diffractometer	9548 independent reflections
Radiation source: fine-focus sealed tube	7113 reflections with *I* > 2σ(*I*)
graphite	*R*_int_ = 0.029
ω scans	θ_max_ = 27.5°, θ_min_ = 1.7°
Absorption correction: multi-scan (*SADABS*; Sheldrick, 1996)	*h* = −15→15
*T*_min_ = 0.667, *T*_max_ = 0.899	*k* = −16→16
19892 measured reflections	*l* = −20→20

### Refinement

**Table:**

Refinement on *F*^2^	Primary atom site location: structure-invariant direct methods
Least-squares matrix: full	Secondary atom site location: difference Fourier map
*R*[*F*^2^ > 2σ(*F*^2^)] = 0.034	Hydrogen site location: inferred from neighbouring sites
*wR*(*F*^2^) = 0.091	H atoms treated by a mixture of independent and constrained refinement
*S* = 1.00	*w* = 1/[σ^2^(*F*_o_^2^) + (0.0461*P*)^2^ + 0.3098*P*] where *P* = (*F*_o_^2^ + 2*F*_c_^2^)/3
9548 reflections	(Δ/σ)_max_ = 0.001
499 parameters	Δρ_max_ = 0.64 e Å^−^^3^
2 restraints	Δρ_min_ = −0.80 e Å^−^^3^

### Fractional atomic coordinates and isotropic or equivalent isotropic displacement parameters (Å^2^)

**Table:**

	*x*	*y*	*z*	*U*_iso_*/*U*_eq_	
Br1	0.82709 (3)	0.56564 (2)	0.965667 (18)	0.03058 (8)	
Br2	0.99128 (3)	0.68807 (2)	0.63996 (2)	0.03598 (9)	
S1	0.39347 (5)	1.31023 (5)	0.49538 (4)	0.02024 (14)	
S2	0.37604 (6)	1.16479 (6)	0.69198 (4)	0.02758 (16)	
S3	1.27628 (5)	−0.14493 (5)	1.10260 (4)	0.01872 (13)	
S4	1.34366 (5)	0.04070 (5)	0.92901 (4)	0.02128 (14)	
O1	0.53444 (15)	1.20078 (15)	0.38665 (11)	0.0229 (4)	
O2	1.05587 (16)	−0.10005 (15)	1.18173 (12)	0.0284 (4)	
N1	0.57049 (19)	0.96785 (18)	0.69482 (13)	0.0196 (4)	
H1	0.508 (2)	1.015 (2)	0.7196 (18)	0.032 (8)*	
N2	1.12817 (19)	0.21396 (17)	0.90374 (13)	0.0188 (4)	
H2	1.2047 (17)	0.180 (2)	0.8909 (19)	0.030 (8)*	
C1	0.5876 (2)	1.1224 (2)	0.44225 (16)	0.0170 (5)	
C2	0.6995 (2)	1.0483 (2)	0.40214 (16)	0.0194 (5)	
H2A	0.7441	1.0971	0.3432	0.023*	
H2B	0.6736	0.9930	0.3860	0.023*	
C3	0.7861 (2)	0.9824 (2)	0.46599 (16)	0.0200 (5)	
C4	0.7094 (2)	0.9213 (2)	0.56034 (16)	0.0203 (5)	
H4A	0.6785	0.8627	0.5523	0.024*	
H4B	0.7623	0.8811	0.6051	0.024*	
C5	0.6032 (2)	0.9988 (2)	0.60205 (16)	0.0164 (5)	
C6	0.5432 (2)	1.1002 (2)	0.54397 (15)	0.0154 (5)	
C7	0.8473 (2)	1.0645 (3)	0.4769 (2)	0.0320 (7)	
H7A	0.7855	1.1283	0.4921	0.048*	
H7B	0.9056	1.0939	0.4182	0.048*	
H7C	0.8899	1.0241	0.5275	0.048*	
C8	0.8822 (2)	0.8941 (2)	0.42616 (18)	0.0303 (6)	
H8A	0.8424	0.8406	0.4208	0.045*	
H8B	0.9374	0.8521	0.4680	0.045*	
H8C	0.9284	0.9329	0.3641	0.045*	
C9	0.4444 (2)	1.1827 (2)	0.57809 (16)	0.0180 (5)	
C10	0.2870 (2)	1.3944 (2)	0.56783 (18)	0.0269 (6)	
H10A	0.2131	1.3611	0.6018	0.032*	
H10B	0.3249	1.3971	0.6141	0.032*	
C11	0.2561 (2)	1.5122 (2)	0.50118 (17)	0.0232 (5)	
C12	0.1633 (2)	1.5403 (2)	0.45648 (19)	0.0289 (6)	
H12	0.1170	1.4848	0.4685	0.035*	
C13	0.1373 (3)	1.6485 (2)	0.3944 (2)	0.0342 (7)	
H13	0.0720	1.6674	0.3653	0.041*	
C14	0.2052 (3)	1.7288 (3)	0.3746 (2)	0.0353 (7)	
H14	0.1883	1.8026	0.3310	0.042*	
C15	0.2980 (3)	1.7010 (2)	0.4186 (2)	0.0355 (7)	
H15	0.3450	1.7564	0.4054	0.043*	
C16	0.3237 (2)	1.5941 (2)	0.48143 (19)	0.0288 (6)	
H16	0.3879	1.5764	0.5114	0.035*	
C17	0.6270 (2)	0.8713 (2)	0.75945 (16)	0.0187 (5)	
C18	0.6361 (2)	0.7603 (2)	0.76156 (17)	0.0224 (5)	
H18	0.6018	0.7469	0.7209	0.027*	
C19	0.6955 (2)	0.6694 (2)	0.82331 (17)	0.0229 (5)	
H19	0.7049	0.5936	0.8235	0.027*	
C20	0.7409 (2)	0.6895 (2)	0.88443 (16)	0.0204 (5)	
C21	0.7265 (2)	0.7985 (2)	0.88659 (17)	0.0239 (6)	
H21	0.7555	0.8108	0.9307	0.029*	
C22	0.6695 (2)	0.8898 (2)	0.82369 (17)	0.0226 (5)	
H22	0.6594	0.9653	0.8245	0.027*	
C23	1.0611 (2)	0.1570 (2)	0.98324 (15)	0.0166 (5)	
C24	0.9302 (2)	0.2136 (2)	1.00927 (16)	0.0193 (5)	
H24A	0.8827	0.1946	0.9776	0.023*	
H24B	0.9252	0.2974	0.9846	0.023*	
C25	0.8708 (2)	0.1806 (2)	1.11405 (16)	0.0194 (5)	
C26	0.8923 (2)	0.0509 (2)	1.14967 (16)	0.0186 (5)	
H26A	0.8603	0.0257	1.2185	0.022*	
H26B	0.8454	0.0251	1.1221	0.022*	
C27	1.0235 (2)	−0.0064 (2)	1.12748 (16)	0.0169 (5)	
C28	0.7351 (2)	0.2307 (2)	1.12632 (18)	0.0275 (6)	
H28A	0.6998	0.1998	1.0949	0.041*	
H28B	0.7233	0.3140	1.0987	0.041*	
H28C	0.6952	0.2104	1.1932	0.041*	
C29	0.9267 (2)	0.2264 (2)	1.16430 (18)	0.0265 (6)	
H29A	1.0128	0.1907	1.1589	0.040*	
H29B	0.8840	0.2085	1.2306	0.040*	
H29C	0.9190	0.3094	1.1356	0.040*	
C30	1.1090 (2)	0.0481 (2)	1.04203 (15)	0.0152 (5)	
C31	1.2339 (2)	−0.0098 (2)	1.02289 (16)	0.0166 (5)	
C32	1.4361 (2)	−0.1873 (2)	1.05017 (17)	0.0214 (5)	
H32A	1.4471	−0.1869	0.9856	0.026*	
H32B	1.4845	−0.1348	1.0482	0.026*	
C33	1.4739 (2)	−0.3061 (2)	1.11252 (17)	0.0207 (5)	
C34	1.4845 (2)	−0.4005 (2)	1.08631 (19)	0.0273 (6)	
H34	1.4704	−0.3896	1.0276	0.033*	
C35	1.5153 (2)	−0.5095 (2)	1.1445 (2)	0.0336 (7)	
H35	1.5237	−0.5731	1.1251	0.040*	
C36	1.5339 (2)	−0.5270 (2)	1.2301 (2)	0.0359 (7)	
H36	1.5540	−0.6024	1.2704	0.043*	
C37	1.5232 (3)	−0.4349 (3)	1.2572 (2)	0.0373 (7)	
H37	1.5354	−0.4466	1.3167	0.045*	
C38	1.4945 (2)	−0.3244 (2)	1.19834 (19)	0.0286 (6)	
H38	1.4891	−0.2611	1.2172	0.034*	
C39	1.0933 (2)	0.3240 (2)	0.84045 (16)	0.0189 (5)	
C40	0.9947 (2)	0.3501 (2)	0.80151 (16)	0.0224 (5)	
H40	0.9484	0.2938	0.8157	0.027*	
C41	0.9646 (2)	0.4589 (2)	0.74193 (17)	0.0238 (6)	
H41	0.8961	0.4787	0.7162	0.029*	
C42	1.0350 (2)	0.5383 (2)	0.72021 (16)	0.0238 (6)	
C43	1.1365 (2)	0.5124 (2)	0.75478 (17)	0.0226 (5)	
H43	1.1855	0.5677	0.7372	0.027*	
C44	1.1655 (2)	0.4037 (2)	0.81590 (16)	0.0217 (5)	
H44	1.2347	0.3838	0.8409	0.026*	

### Atomic displacement parameters (Å^2^)

**Table:**

	*U*^11^	*U*^22^	*U*^33^	*U*^12^	*U*^13^	*U*^23^
Br1	0.03315 (16)	0.02846 (16)	0.02383 (14)	0.00095 (11)	−0.01404 (11)	−0.00052 (11)
Br2	0.04175 (18)	0.01728 (14)	0.02988 (15)	0.00188 (12)	−0.00511 (12)	0.00474 (12)
S1	0.0217 (3)	0.0171 (3)	0.0186 (3)	0.0022 (2)	−0.0048 (2)	−0.0066 (3)
S2	0.0303 (4)	0.0259 (4)	0.0159 (3)	0.0059 (3)	−0.0007 (3)	−0.0072 (3)
S3	0.0171 (3)	0.0149 (3)	0.0184 (3)	−0.0002 (2)	−0.0038 (2)	−0.0014 (2)
S4	0.0167 (3)	0.0201 (3)	0.0189 (3)	−0.0025 (2)	−0.0011 (2)	−0.0008 (3)
O1	0.0242 (9)	0.0226 (9)	0.0157 (8)	0.0037 (7)	−0.0061 (7)	−0.0043 (7)
O2	0.0243 (10)	0.0178 (9)	0.0251 (9)	−0.0005 (7)	0.0015 (7)	0.0040 (8)
N1	0.0219 (11)	0.0185 (11)	0.0143 (10)	0.0002 (9)	−0.0038 (8)	−0.0040 (9)
N2	0.0187 (11)	0.0166 (11)	0.0156 (10)	−0.0005 (8)	−0.0034 (8)	−0.0018 (8)
C1	0.0174 (12)	0.0163 (12)	0.0159 (11)	−0.0036 (9)	−0.0040 (9)	−0.0034 (10)
C2	0.0171 (12)	0.0215 (13)	0.0139 (11)	−0.0021 (10)	−0.0006 (9)	−0.0032 (10)
C3	0.0151 (12)	0.0207 (13)	0.0162 (11)	0.0012 (10)	−0.0022 (9)	−0.0017 (10)
C4	0.0186 (12)	0.0196 (13)	0.0154 (11)	0.0007 (10)	−0.0019 (9)	−0.0026 (10)
C5	0.0163 (12)	0.0170 (12)	0.0154 (11)	−0.0035 (9)	−0.0033 (9)	−0.0048 (10)
C6	0.0132 (11)	0.0178 (12)	0.0135 (11)	−0.0015 (9)	−0.0022 (9)	−0.0051 (10)
C7	0.0195 (14)	0.0412 (17)	0.0360 (16)	−0.0087 (12)	−0.0041 (12)	−0.0127 (14)
C8	0.0247 (14)	0.0311 (15)	0.0186 (13)	0.0080 (11)	−0.0013 (11)	−0.0030 (12)
C9	0.0198 (12)	0.0182 (12)	0.0155 (11)	−0.0019 (9)	−0.0056 (9)	−0.0050 (10)
C10	0.0285 (15)	0.0233 (14)	0.0247 (13)	0.0044 (11)	−0.0043 (11)	−0.0116 (12)
C11	0.0248 (13)	0.0195 (13)	0.0228 (13)	0.0046 (10)	−0.0046 (10)	−0.0111 (11)
C12	0.0299 (15)	0.0241 (14)	0.0367 (16)	0.0000 (11)	−0.0115 (12)	−0.0146 (13)
C13	0.0361 (17)	0.0325 (16)	0.0385 (16)	0.0127 (13)	−0.0207 (13)	−0.0204 (14)
C14	0.0442 (18)	0.0254 (15)	0.0269 (15)	0.0059 (13)	−0.0054 (13)	−0.0098 (13)
C15	0.0333 (16)	0.0246 (15)	0.0466 (18)	−0.0061 (12)	0.0009 (14)	−0.0172 (14)
C16	0.0237 (14)	0.0288 (15)	0.0363 (15)	−0.0004 (11)	−0.0071 (12)	−0.0161 (13)
C17	0.0155 (12)	0.0209 (13)	0.0131 (11)	−0.0010 (10)	−0.0016 (9)	−0.0017 (10)
C18	0.0260 (14)	0.0257 (14)	0.0173 (12)	−0.0080 (11)	−0.0071 (10)	−0.0047 (11)
C19	0.0281 (14)	0.0187 (13)	0.0196 (12)	−0.0049 (10)	−0.0054 (10)	−0.0035 (11)
C20	0.0170 (12)	0.0251 (14)	0.0137 (11)	−0.0019 (10)	−0.0051 (9)	−0.0006 (10)
C21	0.0271 (14)	0.0284 (14)	0.0185 (12)	−0.0035 (11)	−0.0086 (10)	−0.0086 (11)
C22	0.0248 (14)	0.0215 (13)	0.0211 (12)	−0.0021 (10)	−0.0057 (10)	−0.0079 (11)
C23	0.0192 (12)	0.0172 (12)	0.0133 (11)	−0.0028 (9)	−0.0031 (9)	−0.0057 (10)
C24	0.0207 (13)	0.0152 (12)	0.0166 (12)	0.0024 (9)	−0.0054 (9)	−0.0027 (10)
C25	0.0204 (12)	0.0180 (12)	0.0153 (11)	0.0002 (10)	−0.0016 (9)	−0.0051 (10)
C26	0.0186 (12)	0.0206 (13)	0.0159 (11)	−0.0045 (10)	−0.0016 (9)	−0.0065 (10)
C27	0.0187 (12)	0.0140 (12)	0.0172 (11)	−0.0026 (9)	−0.0029 (9)	−0.0055 (10)
C28	0.0237 (14)	0.0247 (14)	0.0233 (13)	0.0037 (11)	−0.0006 (11)	−0.0062 (12)
C29	0.0298 (15)	0.0269 (15)	0.0236 (13)	−0.0066 (11)	0.0001 (11)	−0.0130 (12)
C30	0.0166 (12)	0.0157 (12)	0.0132 (11)	−0.0031 (9)	−0.0024 (9)	−0.0053 (9)
C31	0.0202 (12)	0.0153 (12)	0.0147 (11)	−0.0037 (9)	−0.0052 (9)	−0.0040 (9)
C32	0.0151 (12)	0.0193 (13)	0.0224 (12)	−0.0010 (10)	−0.0021 (10)	−0.0023 (11)
C33	0.0122 (12)	0.0204 (13)	0.0237 (13)	0.0001 (9)	−0.0016 (10)	−0.0052 (11)
C34	0.0213 (13)	0.0271 (15)	0.0301 (14)	0.0003 (11)	−0.0049 (11)	−0.0099 (12)
C35	0.0258 (15)	0.0224 (15)	0.0451 (18)	−0.0013 (11)	−0.0024 (13)	−0.0100 (13)
C36	0.0258 (15)	0.0192 (14)	0.0414 (17)	0.0010 (11)	−0.0025 (13)	0.0051 (13)
C37	0.0326 (16)	0.0418 (18)	0.0252 (14)	0.0048 (13)	−0.0112 (12)	−0.0024 (13)
C38	0.0272 (15)	0.0266 (15)	0.0316 (15)	0.0006 (11)	−0.0116 (12)	−0.0092 (12)
C39	0.0229 (13)	0.0159 (12)	0.0118 (11)	−0.0010 (10)	−0.0018 (9)	−0.0017 (10)
C40	0.0298 (14)	0.0181 (13)	0.0198 (12)	−0.0050 (11)	−0.0063 (10)	−0.0059 (11)
C41	0.0274 (14)	0.0223 (14)	0.0177 (12)	0.0001 (11)	−0.0063 (10)	−0.0047 (11)
C42	0.0320 (15)	0.0139 (12)	0.0143 (11)	0.0017 (10)	−0.0019 (10)	0.0004 (10)
C43	0.0274 (14)	0.0164 (13)	0.0190 (12)	−0.0059 (10)	0.0000 (10)	−0.0032 (10)
C44	0.0197 (13)	0.0229 (13)	0.0181 (12)	−0.0024 (10)	−0.0013 (10)	−0.0057 (11)

### Geometric parameters (Å, °)

**Table:**

Br1—C20	1.895 (2)	C18—C19	1.385 (3)
Br2—C42	1.896 (2)	C18—H18	0.9500
S1—C9	1.756 (2)	C19—C20	1.378 (3)
S1—C10	1.824 (2)	C19—H19	0.9500
S2—C9	1.685 (2)	C20—C21	1.378 (4)
S3—C31	1.765 (2)	C21—C22	1.385 (3)
S3—C32	1.820 (2)	C21—H21	0.9500
S4—C31	1.687 (2)	C22—H22	0.9500
O1—C1	1.227 (3)	C23—C30	1.431 (3)
O2—C27	1.225 (3)	C23—C24	1.506 (3)
N1—C5	1.326 (3)	C24—C25	1.528 (3)
N1—C17	1.427 (3)	C24—H24A	0.9900
N1—H1	0.90 (2)	C24—H24B	0.9900
N2—C23	1.325 (3)	C25—C26	1.519 (3)
N2—C39	1.427 (3)	C25—C28	1.525 (3)
N2—H2	0.88 (2)	C25—C29	1.530 (3)
C1—C6	1.471 (3)	C26—C27	1.506 (3)
C1—C2	1.506 (3)	C26—H26A	0.9900
C2—C3	1.518 (3)	C26—H26B	0.9900
C2—H2A	0.9900	C27—C30	1.472 (3)
C2—H2B	0.9900	C28—H28A	0.9800
C3—C8	1.527 (3)	C28—H28B	0.9800
C3—C7	1.528 (4)	C28—H28C	0.9800
C3—C4	1.529 (3)	C29—H29A	0.9800
C4—C5	1.505 (3)	C29—H29B	0.9800
C4—H4A	0.9900	C29—H29C	0.9800
C4—H4B	0.9900	C30—C31	1.443 (3)
C5—C6	1.425 (3)	C32—C33	1.504 (3)
C6—C9	1.452 (3)	C32—H32A	0.9900
C7—H7A	0.9800	C32—H32B	0.9900
C7—H7B	0.9800	C33—C38	1.381 (4)
C7—H7C	0.9800	C33—C34	1.390 (4)
C8—H8A	0.9800	C34—C35	1.376 (4)
C8—H8B	0.9800	C34—H34	0.9500
C8—H8C	0.9800	C35—C36	1.370 (4)
C10—C11	1.505 (3)	C35—H35	0.9500
C10—H10A	0.9900	C36—C37	1.371 (4)
C10—H10B	0.9900	C36—H36	0.9500
C11—C12	1.382 (4)	C37—C38	1.389 (4)
C11—C16	1.389 (4)	C37—H37	0.9500
C12—C13	1.384 (4)	C38—H38	0.9500
C12—H12	0.9500	C39—C44	1.387 (3)
C13—C14	1.372 (4)	C39—C40	1.389 (3)
C13—H13	0.9500	C40—C41	1.381 (3)
C14—C15	1.376 (4)	C40—H40	0.9500
C14—H14	0.9500	C41—C42	1.378 (4)
C15—C16	1.376 (4)	C41—H41	0.9500
C15—H15	0.9500	C42—C43	1.380 (4)
C16—H16	0.9500	C43—C44	1.388 (3)
C17—C22	1.389 (3)	C43—H43	0.9500
C17—C18	1.392 (3)	C44—H44	0.9500
			
C9—S1—C10	103.16 (11)	C22—C21—H21	120.4
C31—S3—C32	103.22 (11)	C21—C22—C17	120.1 (2)
C5—N1—C17	127.3 (2)	C21—C22—H22	119.9
C5—N1—H1	116.3 (18)	C17—C22—H22	119.9
C17—N1—H1	116.3 (18)	N2—C23—C30	122.5 (2)
C23—N2—C39	128.3 (2)	N2—C23—C24	116.2 (2)
C23—N2—H2	114.4 (18)	C30—C23—C24	121.2 (2)
C39—N2—H2	117.3 (18)	C23—C24—C25	115.47 (19)
O1—C1—C6	121.6 (2)	C23—C24—H24A	108.4
O1—C1—C2	117.2 (2)	C25—C24—H24A	108.4
C6—C1—C2	121.1 (2)	C23—C24—H24B	108.4
C1—C2—C3	115.15 (19)	C25—C24—H24B	108.4
C1—C2—H2A	108.5	H24A—C24—H24B	107.5
C3—C2—H2A	108.5	C26—C25—C28	110.6 (2)
C1—C2—H2B	108.5	C26—C25—C24	106.01 (19)
C3—C2—H2B	108.5	C28—C25—C24	108.15 (19)
H2A—C2—H2B	107.5	C26—C25—C29	111.1 (2)
C2—C3—C8	110.2 (2)	C28—C25—C29	110.0 (2)
C2—C3—C7	110.0 (2)	C24—C25—C29	110.8 (2)
C8—C3—C7	110.0 (2)	C27—C26—C25	114.4 (2)
C2—C3—C4	106.50 (19)	C27—C26—H26A	108.6
C8—C3—C4	109.4 (2)	C25—C26—H26A	108.6
C7—C3—C4	110.6 (2)	C27—C26—H26B	108.6
C5—C4—C3	114.3 (2)	C25—C26—H26B	108.6
C5—C4—H4A	108.7	H26A—C26—H26B	107.6
C3—C4—H4A	108.7	O2—C27—C30	121.4 (2)
C5—C4—H4B	108.7	O2—C27—C26	117.9 (2)
C3—C4—H4B	108.7	C30—C27—C26	120.6 (2)
H4A—C4—H4B	107.6	C25—C28—H28A	109.5
N1—C5—C6	122.6 (2)	C25—C28—H28B	109.5
N1—C5—C4	116.3 (2)	H28A—C28—H28B	109.5
C6—C5—C4	121.1 (2)	C25—C28—H28C	109.5
C5—C6—C9	124.8 (2)	H28A—C28—H28C	109.5
C5—C6—C1	116.6 (2)	H28B—C28—H28C	109.5
C9—C6—C1	118.6 (2)	C25—C29—H29A	109.5
C3—C7—H7A	109.5	C25—C29—H29B	109.5
C3—C7—H7B	109.5	H29A—C29—H29B	109.5
H7A—C7—H7B	109.5	C25—C29—H29C	109.5
C3—C7—H7C	109.5	H29A—C29—H29C	109.5
H7A—C7—H7C	109.5	H29B—C29—H29C	109.5
H7B—C7—H7C	109.5	C23—C30—C31	124.3 (2)
C3—C8—H8A	109.5	C23—C30—C27	116.7 (2)
C3—C8—H8B	109.5	C31—C30—C27	119.0 (2)
H8A—C8—H8B	109.5	C30—C31—S4	125.17 (18)
C3—C8—H8C	109.5	C30—C31—S3	118.00 (17)
H8A—C8—H8C	109.5	S4—C31—S3	116.82 (13)
H8B—C8—H8C	109.5	C33—C32—S3	105.03 (16)
C6—C9—S2	124.35 (18)	C33—C32—H32A	110.7
C6—C9—S1	117.94 (17)	S3—C32—H32A	110.7
S2—C9—S1	117.71 (14)	C33—C32—H32B	110.7
C11—C10—S1	105.70 (17)	S3—C32—H32B	110.7
C11—C10—H10A	110.6	H32A—C32—H32B	108.8
S1—C10—H10A	110.6	C38—C33—C34	118.4 (2)
C11—C10—H10B	110.6	C38—C33—C32	121.1 (2)
S1—C10—H10B	110.6	C34—C33—C32	120.5 (2)
H10A—C10—H10B	108.7	C35—C34—C33	120.7 (3)
C12—C11—C16	118.7 (2)	C35—C34—H34	119.7
C12—C11—C10	121.4 (3)	C33—C34—H34	119.7
C16—C11—C10	119.9 (2)	C36—C35—C34	120.6 (3)
C11—C12—C13	120.6 (3)	C36—C35—H35	119.7
C11—C12—H12	119.7	C34—C35—H35	119.7
C13—C12—H12	119.7	C35—C36—C37	119.5 (3)
C14—C13—C12	120.4 (3)	C35—C36—H36	120.3
C14—C13—H13	119.8	C37—C36—H36	120.3
C12—C13—H13	119.8	C36—C37—C38	120.5 (3)
C13—C14—C15	119.2 (3)	C36—C37—H37	119.8
C13—C14—H14	120.4	C38—C37—H37	119.8
C15—C14—H14	120.4	C33—C38—C37	120.4 (3)
C14—C15—C16	120.9 (3)	C33—C38—H38	119.8
C14—C15—H15	119.5	C37—C38—H38	119.8
C16—C15—H15	119.5	C44—C39—C40	120.7 (2)
C15—C16—C11	120.2 (3)	C44—C39—N2	117.0 (2)
C15—C16—H16	119.9	C40—C39—N2	122.2 (2)
C11—C16—H16	119.9	C41—C40—C39	119.3 (2)
C22—C17—C18	119.9 (2)	C41—C40—H40	120.4
C22—C17—N1	118.4 (2)	C39—C40—H40	120.4
C18—C17—N1	121.6 (2)	C42—C41—C40	119.4 (2)
C19—C18—C17	119.6 (2)	C42—C41—H41	120.3
C19—C18—H18	120.2	C40—C41—H41	120.3
C17—C18—H18	120.2	C41—C42—C43	122.2 (2)
C20—C19—C18	119.7 (2)	C41—C42—Br2	119.00 (19)
C20—C19—H19	120.1	C43—C42—Br2	118.8 (2)
C18—C19—H19	120.1	C42—C43—C44	118.4 (2)
C21—C20—C19	121.3 (2)	C42—C43—H43	120.8
C21—C20—Br1	119.17 (18)	C44—C43—H43	120.8
C19—C20—Br1	119.56 (19)	C39—C44—C43	120.0 (2)
C20—C21—C22	119.2 (2)	C39—C44—H44	120.0
C20—C21—H21	120.4	C43—C44—H44	120.0
			
O1—C1—C2—C3	159.1 (2)	C39—N2—C23—C30	−177.8 (2)
C6—C1—C2—C3	−21.9 (3)	C39—N2—C23—C24	1.9 (4)
C1—C2—C3—C8	169.2 (2)	N2—C23—C24—C25	−153.0 (2)
C1—C2—C3—C7	−69.3 (3)	C30—C23—C24—C25	26.7 (3)
C1—C2—C3—C4	50.6 (3)	C23—C24—C25—C26	−52.7 (3)
C2—C3—C4—C5	−55.0 (3)	C23—C24—C25—C28	−171.4 (2)
C8—C3—C4—C5	−174.1 (2)	C23—C24—C25—C29	68.0 (3)
C7—C3—C4—C5	64.6 (3)	C28—C25—C26—C27	171.30 (19)
C17—N1—C5—C6	−177.1 (2)	C24—C25—C26—C27	54.3 (3)
C17—N1—C5—C4	2.2 (4)	C29—C25—C26—C27	−66.1 (3)
C3—C4—C5—N1	−148.8 (2)	C25—C26—C27—O2	150.5 (2)
C3—C4—C5—C6	30.5 (3)	C25—C26—C27—C30	−30.8 (3)
N1—C5—C6—C9	2.6 (4)	N2—C23—C30—C31	1.4 (4)
C4—C5—C6—C9	−176.7 (2)	C24—C23—C30—C31	−178.3 (2)
N1—C5—C6—C1	−178.7 (2)	N2—C23—C30—C27	−178.9 (2)
C4—C5—C6—C1	2.1 (3)	C24—C23—C30—C27	1.3 (3)
O1—C1—C6—C5	172.4 (2)	O2—C27—C30—C23	179.4 (2)
C2—C1—C6—C5	−6.5 (3)	C26—C27—C30—C23	0.7 (3)
O1—C1—C6—C9	−8.7 (3)	O2—C27—C30—C31	−0.9 (3)
C2—C1—C6—C9	172.3 (2)	C26—C27—C30—C31	−179.7 (2)
C5—C6—C9—S2	−6.4 (3)	C23—C30—C31—S4	0.1 (3)
C1—C6—C9—S2	174.91 (18)	C27—C30—C31—S4	−179.54 (17)
C5—C6—C9—S1	173.03 (18)	C23—C30—C31—S3	−179.07 (18)
C1—C6—C9—S1	−5.7 (3)	C27—C30—C31—S3	1.3 (3)
C10—S1—C9—C6	−172.39 (19)	C32—S3—C31—C30	178.16 (18)
C10—S1—C9—S2	7.04 (18)	C32—S3—C31—S4	−1.10 (17)
C9—S1—C10—C11	170.72 (18)	C31—S3—C32—C33	−176.12 (17)
S1—C10—C11—C12	86.5 (3)	S3—C32—C33—C38	−76.0 (3)
S1—C10—C11—C16	−91.5 (2)	S3—C32—C33—C34	101.5 (2)
C16—C11—C12—C13	−1.0 (4)	C38—C33—C34—C35	−0.2 (4)
C10—C11—C12—C13	−179.1 (2)	C32—C33—C34—C35	−177.9 (2)
C11—C12—C13—C14	1.6 (4)	C33—C34—C35—C36	1.2 (4)
C12—C13—C14—C15	−1.2 (4)	C34—C35—C36—C37	−0.8 (4)
C13—C14—C15—C16	0.3 (4)	C35—C36—C37—C38	−0.4 (4)
C14—C15—C16—C11	0.2 (4)	C34—C33—C38—C37	−1.0 (4)
C12—C11—C16—C15	0.1 (4)	C32—C33—C38—C37	176.6 (2)
C10—C11—C16—C15	178.2 (2)	C36—C37—C38—C33	1.4 (4)
C5—N1—C17—C22	122.2 (3)	C23—N2—C39—C44	127.2 (3)
C5—N1—C17—C18	−60.3 (3)	C23—N2—C39—C40	−55.4 (3)
C22—C17—C18—C19	−4.7 (4)	C44—C39—C40—C41	−3.7 (4)
N1—C17—C18—C19	177.8 (2)	N2—C39—C40—C41	178.9 (2)
C17—C18—C19—C20	2.5 (4)	C39—C40—C41—C42	1.8 (4)
C18—C19—C20—C21	1.1 (4)	C40—C41—C42—C43	1.2 (4)
C18—C19—C20—Br1	−177.76 (18)	C40—C41—C42—Br2	−178.97 (18)
C19—C20—C21—C22	−2.5 (4)	C41—C42—C43—C44	−2.2 (4)
Br1—C20—C21—C22	176.43 (18)	Br2—C42—C43—C44	177.91 (18)
C20—C21—C22—C17	0.2 (4)	C40—C39—C44—C43	2.7 (4)
C18—C17—C22—C21	3.4 (4)	N2—C39—C44—C43	−179.9 (2)
N1—C17—C22—C21	−179.0 (2)	C42—C43—C44—C39	0.3 (4)

### Hydrogen-bond geometry (Å, °)

**Table:**

*D*—H···*A*	*D*—H	H···*A*	*D*···*A*	*D*—H···*A*
N1—H1···S2	0.90 (2)	2.11 (2)	2.890 (2)	146 (2)
N2—H2···S4	0.88 (2)	2.10 (2)	2.887 (2)	149 (2)
